# Retraction: Anti-apoptosis mechanism of triptolide based on network pharmacology in focal segmental glomerulosclerosis rats

**DOI:** 10.1042/BSR-20192920_RET

**Published:** 2020-09-29

**Keywords:** apoptosis, FSGS, IL4, network pharmacology, triptolide

This article is being retracted from Bioscience Reports at the request of the authors.

The authors have been unable to replicate the results produced in the study, in particular the quantity of podocytes undergoing apoptosis following treatment with Triptolide (TPL), causing the authors to doubt the original conclusion that TPL has no influence on the cell viability and apoptosis. The authors are still investigating the mechanism of TPL regulation on podocyte lesions and the reason for this discrepancy is currently unknown.

The authors also note that there are errors in the units throughout the paper, as well as a “Glucose treatment and cell culture” section in the methods, which did not form part of this study. The Editorial Board agrees to the retraction.

